# Endoscopic Approaches to the Treatment of Variceal Hemorrhage in Hemodialysis-Dependent Patients

**DOI:** 10.1155/2016/9732039

**Published:** 2016-12-26

**Authors:** Xiaoquan Huang, Lili Ma, Xiaoqing Zeng, Jian Wang, Jie Chen, Shiyao Chen

**Affiliations:** ^1^Endoscopy Center and Endoscopy Research Institute, Zhongshan Hospital, Fudan University, Shanghai 200032, China; ^2^Department of Gastroenterology, Zhongshan Hospital, Fudan University, Shanghai 200032, China

## Abstract

*Background*. Esophagogastric variceal hemorrhage leads to challenging situation in chronic kidney disease patients on maintenance hemodialysis.* Aims*. To determine the safety and efficacy of endoscopic approaches to patients with hemodialysis-dependent concomitant with esophagogastric varices.* Methods*. Medical records were reviewed from January 1, 2004, to December 31, 2015, in our hospital. Five consecutive hemodialysis-dependent patients with variceal hemorrhage who underwent endoscopic treatments were retrospectively studied.* Results*. The median age of the patients was 54 years (range 34–67 years) and the median follow-up period was 21.3 months (range 7–134 months). All the patients received a total of three times heparin-free hemodialysis 24 hours before and no more than 24 hours and 72 hours after endoscopic treatment. They successfully had endoscopic variceal ligation, endoscopic injection sclerotherapy, and/or N-butyl cyanoacrylate injection. The short-term efficacy is satisfying and long-term follow-up showed episodes of rebleeding.* Conclusions*. Endoscopic approaches are the alternative options in the treatment of upper gastroenterology variceal hemorrhage in hemodialysis-dependent patients without severe complications.

## 1. Introduction

The global prevalence and incidence of end-stage renal disease (ESRD) are increasing worldwide while the mortality among patients with ESRD observed large net reductions due to both dialysis and transplantation [1]. Cirrhotic patients with ESRD have increased complications and higher mortality rate [2]. The prevalence of cirrhosis was about 6.2% at the beginning of dialysis in a cohort study in Taiwan [3]. Esophagogastric variceal bleeding accounts for a mortality rate of approximately 15% in general population [4]. Evidence had proven endoscopic approaches are the most effective intervention for primary and secondary prevention of gastroesophageal variceal bleeding, recommended as the first-line option [5].

However, patients with marked impaired renal function undergoing hemodialysis and concomitant cirrhotic gastroesophageal varices remained intractable. Their fragile hemostasis states, caused by renal anemia, uremic platelet dysfunction, and use of anticoagulants [6, 7], lead to recurrent upper gastroenterology bleeding. Haskal and Radhakrishnan [8] had reported that transjugular intrahepatic portosystemic shunts (TIPS) were effective in controlling ascites and bleeding in dialysis-dependent patients but a high incidence of post-TIPS hepatic encephalopathy. Simultaneous liver-kidney transplantation (SLKT) is the best choice for them considering both survival and quality-adjusted life years [9, 10]. However, it takes time waiting for the donor and some patients died of massive variceal hemorrhage during the waiting period. Endoscopic treatment might be the alternative option to avoid massive variceal bleeding. The concerns of the endoscopic approach are the risk of bleeding and anesthesia management. To our knowledge, no literature reporting endoscopic treatment of varices in hemodialysis-dependent patients was found. In this study, we report our experiences in controlling esophagogastric variceal bleeding in hemodialysis-dependent patients.

## 2. Materials and Methods

### 2.1. Study Design

In this tertiary hospital-based retrospective case series study, a total of 2038 consecutive hospitalized patients with esophageal and/or gastric varices undergoing endoscopic therapy were screened. We included (1) patients who required maintenance hemodialysis; (2) patients confirmed to have liver cirrhosis by computed tomography; (3) patients confirmed to have varices by gastroscopy; and (4) patients who had endoscopic treatment for secondary prevention of variceal bleeding. We excluded patients who had endoscopic treatment before starting hemodialysis. We retrieved medical records including the emergency room and outpatient department from our hospital between January 1, 2004, and December 31, 2015. The end point was set at March 31, 2016. All the patients were followed up via phone calls and outpatient clinic visits. This retrospective report was approved by the institution's Ethics Committee and written informed consent was obtained from each patient. Median and range were shown to describe quantitative data.

### 2.2. Endoscopic Intervention

The devices and drugs used included the electronic endoscope GIF-XQ240/260 (Olympus, Tokyo, Japan), 6 multiband ligators (Cook Endoscopy, Winston-Salem, North Carolina, USA) or 7 multiband ligators (Boston Scientific, Natick, Massachusetts, USA), N-butyl-cyanoacrylate (Beijing Suncon Medical Adhesive, Beijing, China), lauromacrogol (Tianyu Pharmaceutical, China), lipiodol, and injection needle (Olympus NM-200 L-423, Tokyo, Japan).

Gastric varices were treated with N-butyl-cyanoacrylate using the sandwich method (lipiodol or 20% glucose or lauromacrogol-cyanoacrylate-lipiodol or 20% glucose or lauromacrogol). Each cyanoacrylate injection point was no more than 2.0 mL and an equal volume of lipiodol or 20% glucose or 2–8 mL lauromacrogol determined by the varix size according to our published study [11]. Multiple sites injection was an attempt to completely obturate the gastric varices in one session. To decrease the risk of a variceal tear, the needle sheath was held in the puncture site to prevent leakage of the cyanoacrylate and to ensure the varice has hardened before retracting the injector catheter.

Endoscopic variceal ligation (EVL) is the primary treatment selection for esophageal varices according to guideline and our experiences [12, 13]. Ligation was applied from 1 cm above the Z-line in a spirally ascending fashion, with no more than six or seven bands used per session. Endoscopic injection sclerosis (EIS) treatment is usually performed in patients who had multiple EVL sessions and as an attempt to eliminate the small esophageal varix. The initial injection started above the Z-line, and intravariceal or paravariceal injection of 10–30 mL per session of lauromacrogol was injected. Follow-up endoscopy was performed at an interval of no less than 2 months and treatment was repeated until complete obliteration was achieved.

### 2.3. Perioperative Management

All the patients required hemodialysis three times per week. Heparin-free dialysis was performed the day before the endoscopic procedure and postoperative heparin-free dialysis was performed no more than 24 hours after the procedures, and the third heparin-free dialysis was done more than 72 hours after the procedure. Patients did not receive any other anticoagulation drugs for a week. Later comes the routine usage of heparin dialysis. They were adequately treated with proton pump inhibitors (PPIs) after endoscopic treatment and no prophylactic antibiotics were used.

### 2.4. Anesthesia Management

The patient's general condition should be designated as American Society of Anesthesiologists (ASA) classification I, II, or III, and these patients underwent propofol sedation for endoscopy [14]. The initial dosage was 2 mg/kg, and 1 mg/kg was added by the anesthesiologist if body movement disturbed the procedure. Electrocardiogram, oxygen saturation, respiratory rate, and noninvasive blood pressure measurements were monitored during the endoscopic treatment.

### 2.5. Statistical Analysis

Statistical analysis was performed with SPSS 23.0 software (SPSS Inc., Chicago, Illinois, USA). Median and range were shown to describe continuous data.

## 3. Results

Five patients were enrolled in this study, and their demographic data were shown in Table 1. Of the five patients, there were three males and two females with the age of 54 years (range, 33–67 years) and the creatinine of 738 *μ*mol/L (range, 542–1131 *μ*mol/L) at the time of having the first endoscopic treatment. The etiologies of liver cirrhosis included hepatic B virus (HBV), hepatic C virus (HCV), and drug-induced-liver-injury (DILI) (aristolochic acid and unknown herb in traditional Chinese medicine). The primary diseases related to renal failure are chronic glomerulonephritis (80%) and aristolochic acid nephropathy (20%). Among these patients, one had hepatocellular carcinoma (HCC, patient 1) and another one had colorectal cancer (CRC, patient 4) before developing into gastroesophageal varices and starting maintenance hemodialysis. The liver function of these patients was mild impairment.

**Figure 1:**
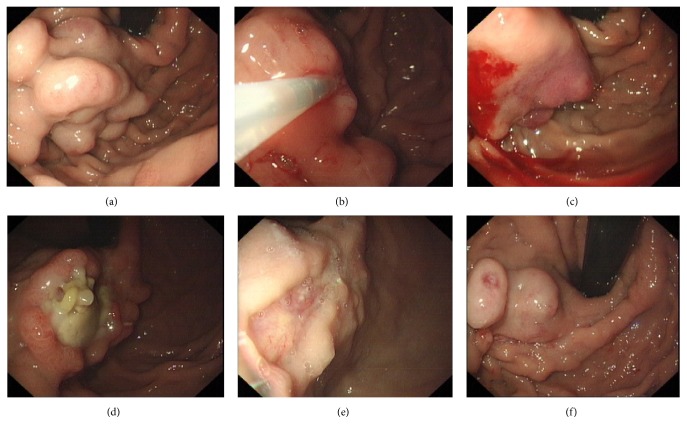
Gastric varices. (a) Isolate gastric varices vein (4 cm) before treatment. (b) Injecting 6 mL lauromacrogol + 1.5 mL N-butyl-cyanoacrylate + 4 mL lauromacrogol. (c) Mild blood exudation after injection; the injection site was rinsed with ice-cold norepinephrine. (d) Gastric ulceration in the injection site. (e) Scar of cyanoacrylate injection. (f) Recurrence of gastric varices in 10 months (patient 2).

**Figure 2:**
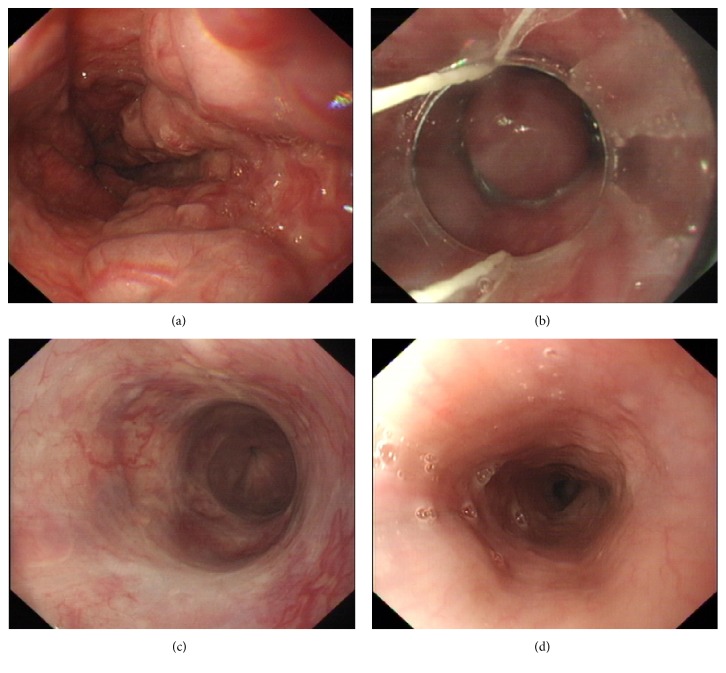
Esophageal varices. (a) Esophageal appearance before treatment. (b) Endoscopic variceal ligations. (c) Esophageal appearance after endoscopic variceal ligation procedures. (d) Brand new esophageal appearance after simultaneous liver-kidney transplantation (patient 1).

Among the five cases, the time interval between hemodialysis and first endoscopic treatment was 37.5 months (range 5.9–94.6 months). A total of 16 endoscopic procedures were performed including 11 EVLs, 2 EISs, and 3 N-butyl cyanoacrylate injections. The details of endoscopic treatment were shown in Table 2. All the procedures were secondary prophylaxis of bleeding. The changes before and after endoscopic treatment of gastric varices were shown in Figure 1. All the three cyanoacrylate injection procedures had immediate blood exudations and were controlled by rinsing with 8% ice norepinephrine solution. The esophageal varices were treated by ligations and sclerotherapy (Figures 2(a), 2(b), and 2(c)). No delay bleeding was observed when they were back to the ward. No complications such as infections, massive bleeding, or stroke were observed in these patients.

Of the five patients, only one (20%) experienced rebleeding within 3 months after the endoscopic treatment and three (60%) suffered from nonlethal rebleeding episodes after 3 months. The median follow-up period was 21.3 months (range 7–134 months). Patient 1 received SLKT in 2013 and completely cured without severe complications (Figure 2(d)).

## 4. Discussion

An increasing number of patients with concurrent ESRD and varices make the management of variceal hemorrhage intractable. The concerns of endoscopic approaches are the risk of bleeding and anesthesia. Our experiences illustrate that endoscopic treatment is effective and relatively safe in these patients. All of our patients' esophageal varices were prominent in the distal esophagus, in contrast to the “downhill varices” in the proximal esophagus in hemodialysis patient with central venous catheters [15]. “Uphill” varices caused by cirrhotic portal hypertension are much easy to bleed. Endoscopic approaches are promising in the hemorrhage management [16].

While the impaired renal and hepatic functions increased the risk of sedation and propofol, an ultrashort acting hypnotic agent was given. Propofol is widely recommended in short endoscopic procedures and superior in terms of patient's tolerance, maximum level of sedation achieved, and shorter recovery room times [17]. Only very little part of propofol clearance is through renal elimination. Its pharmacokinetics are minimally changed in patients with impaired liver and renal functions [18]. Patients sedated with propofol were confirmed to be safe and less invasive by a large amount of patients undergoing endoscopic treatments in our hospital. Closely monitoring the blood pressure and limitation of fluid infusion are required.

Because none of our patients had endoscopic procedures for the management of acute variceal hemorrhage, propofol sedation is possible in secondary prophylaxis for variceal hemorrhage. The sedation helps with the compliable of patients during treatments and reduces the possibility of bleeding and makes bleeding easy to control during injection. Extravascular injection of drugs is easy to cause gastric ulceration and precise intravascular injection can also reduce rebleeding risk [19]. In addition, the administration of propofol was by anesthesiologists, and we can immediately switch into intubation when necessary.

Little is known about the safety of long-term heparin-free hemodialysis; however, temporarily heparin-free hemodialysis without anticoagulation drug is beneficial to these patients for endoscopic procedures. Perioperative heparin-free dialysis reduced the risk of bleeding during endoscopic treatment and delay bleeding.

Long-term rebleeding episodes might be because of the high portal pressure in these patients. However, because of their poor general conditions, none of them had hepatic venous pressure gradient (HVPG) measurement. The actual pressure of these patients remained unknown. Further study will be required to evaluate the long-term outcomes in more patients.

In conclusion, for hemodialysis-dependent patients with esophagogastric varices, endoscopic approaches might be the alternative options to TIPS and SKLT, which reduce the risk of variceal hemorrhage without severe complications.

## Figures and Tables

**Table 1:** Demographic data in five patients undergoing endoscopic treatment.

Patient number	Gender	Age	Etiology of cirrhosis	Primary renal disease	Creatinine (*μ*mol/L)	eGFR (ml/min/1.73 m^2^)	Time interval between hemodialysis start and first bleeding (months)	Varices type	Child-Pugh grade	Concomitant tumor
1	Male	34	HBV	Chronic glomerulonephritis	738	7.4	3.2	Only EV (G3)	B	HCC
2	Male	51	HCV	Chronic glomerulonephritis	878	5.6	34.9	Only GV (IGV)	B	/
3	Male	54	HBV	Chronic glomerulonephritis	1131	4.1	105.1	Only EV (G3)	B	/
4	Female	64	DILI^*∗*^	Chronic glomerulonephritis	546	6.8	12.6	Both EV (G3) and GV (GOV-2)	A	CRC
5	Female	67	DILI^†^	Aristolochic acid nephropathy	542	7.4	81.9	Both EV (G3) and GV (GOV-1)	B	/

HBV, hepatitis B virus; HCV, hepatitis C virus; HCC, hepatocellular carcinoma; CRC, colorectal cancer; DILI, drug-induced liver injury.

eGFR, estimate glomerular filtrate rate, using CKD-EPI (Chronic Kidney Disease Epidemiology Collaboration) equation.

Esophageal varices, Grade 3 (EV, G3), were defined as large, coil-shaped EV occupying more than one-third of the lumen.

Gastric varices (GV) were defined according to Sarin's classification as lesser curvature varices (gastroesophageal varices type 1, GOV-1), greater curvature varices (GOV-2), or isolated gastric varices type (IGV).

^*∗*^Induced by unknown herb in traditional Chinese medicine.

^†^Induced by aristolochic acid.

**Table 2:** Endoscopic treatment details of hemodialysis patients.

Patient number	Time interval between first bleeding and treatment (months)	Time interval^*∗*^(months)	Treatment options*∗*numbers	Total treatments	Rebleeding (within 3 months)	Rebleeding (after 3 months)	Follow-up (months)	Time interval between the end of treatment and death (months)	Outcome
1	2.7	5.9	EVL*∗*4 and EIS*∗*1	5	Yes	Yes	64.4	/	SLKT in 2013/alive
2	2.6	37.5	NBCA 1.5 ml and NBCA 0.5 ml	2	No	Yes	10.2	3.8	Die in May, 2014
3	1.7	94.6	EVL*∗*5 and EIS*∗*1	6	No	Yes	134.2	/	Alive
4	1.8	14.4	NBCA 4ml and EVL*∗*1	2	No	No	7.7	/	Alive
5	8.0	89.9	EVL*∗*1	1	No	No	21.3	21.7	Die in Aug, 2013

EVL, endoscopic variceal ligation; EIS, endoscopic injection sclerosis; NBCA, N-butyl-cyanoacrylate; SLKT, simultaneous liver-kidney transplantation.

^*∗*^Time interval: the interval between hemodialysis start and first time receiving endoscopic treatment.
